# In vitro and in vivo tenocyte-protective effectiveness of dehydroepiandrosterone against high glucose-induced oxidative stress

**DOI:** 10.1186/s12891-021-04398-z

**Published:** 2021-06-05

**Authors:** Shintaro Mukohara, Yutaka Mifune, Atsuyuki Inui, Hanako Nishimoto, Takashi Kurosawa, Kohei Yamaura, Tomoya Yoshikawa, Ryosuke Kuroda

**Affiliations:** Department of Orthopaedic Surgery, Kobe University Graduate School of Medicine, 7-5-2 Kusunoki-cho, Chuo-ku, 650-0017 Kobe, Japan

**Keywords:** High glucose, Oxidative stress, Dehydroepiandrosterone, NADPH oxidase, Diabetic tendinopathy

## Abstract

**Background:**

Dehydroepiandrosterone (DHEA), an adrenal steroid, has a protective role against diabetes. This study aimed to investigate the in vitro and in vivo protective effects of DHEA against high glucose-induced oxidative stress in tenocytes and tendons.

**Methods:**

Tenocytes from normal Sprague-Dawley rats were cultured in low-glucose (LG) or high-glucose (HG) medium with or without DHEA. The experimental groups were: control group (LG without DHEA), LG with DHEA, HG without DHEA, and HG with DHEA. Reactive oxygen species (ROS) production, apoptosis, and messenger RNA (mRNA) expression of NADPH oxidase (NOX) 1 and 4, and interleukin-6 (IL-6) were determined. Further, diabetic rats were divided into a control group and a DHEA-injected group (DHEA group). NOX1 and NOX4 protein expression and mRNA expression of NOX1, NOX4, IL-6, matrix metalloproteinase (MMP)-2, tissue inhibitors of matrix metalloproteinase (TIMP)-2, and type I and III collagens in the Achilles tendon were determined.

**Results:**

In rat tenocytes, DHEA decreased the expression of NOX1 and IL-6, ROS accumulation, and apoptotic cells. In the diabetic rat Achilles tendon, NOX1 protein expression and mRNA expression of NOX1, IL-6, MMP-2, TIMP-2, and type III collagen were significantly lower while type I collagen expression was significantly higher in the DHEA group than in the control group.

**Conclusions:**

DHEA showed antioxidant and anti-inflammatory effects both in vitro and in vivo. Moreover, DHEA improved tendon matrix synthesis and turnover, which are affected by hyperglycemic conditions. DHEA is a potential preventive drug for diabetic tendinopathy.

## Background

Musculoskeletal disorders such as tendinitis [1], Dupuytren’s disease [2], carpal tunnel syndrome [3], adhesive capsulitis [4], and calcific tendinopathy [5] are more common in patients with diabetes mellitus (DM). Since DM causes structural, inflammatory, and vascular changes in tendons, DM is considered an important risk/causative factor associated with the development of chronic tendinopathy [6, 7]. In addition, the presence and severity of DM have been reported to generally aggravate tendinopathy and tendon rupture and contribute to poor prognosis. For example, the degree of Achilles tendon degeneration and shoulder pain and stiffness associated with rotator cuff tears are more severe in patients with DM [8, 9]. Although the pathogenesis of diabetic tendinopathy is still not fully understood, the potential mechanisms by which DM causes and exacerbates tendinopathy are – accumulation of advanced glycation end-products (AGEs), adipokine dysregulation, increased reactive oxygen species (ROS), cell death and inflammatory cytokines, imbalance in the matrix metalloproteinase (MMP)/tissue inhibitor (TIMP) ratio, and impaired angiogenesis and tendon sheath differentiation [6]. While diabetic tendinopathy can be caused by multiple factors, increased ROS has been suggested to be one of the important pathogenic mechanisms of tendon degeneration in chronic tendinopathy [6].

A number of studies have demonstrated that hyperglycemic conditions induce oxidative stress and cytokine production, and oxidative stress increases the production of ROS, causing inflammation, cell death, and tissue damage in various types of cells [10–13]. The main pathways of ROS production in hyperglycemia have been reported, such as NADPH oxidase (NOX) [14], the accumulation of AGEs [15], and the increase in superoxide production by the mitochondrial electron transfer system [16].

NOX, one of the main ROS generating pathways, is a family of multi-subunit enzymes located on the cell membrane and activated by protein kinase C to produce ROS [14]. Under pathological conditions, the upregulation of tissue- and disease-specific NOX subtypes can cause overproduction of superoxide, a type of ROS [17]. Previous reports showed NOX1 expression was increased in rat tenocytes and Achilles tendons under hyperglycemic conditions, and the accompanying ROS overproduction and inflammatory reaction contributed to diabetic tendinopathy [18, 19].

Dehydroepiandrosterone (DHEA) is the most abundant circulating steroid hormone produced by the mammalian adrenal cortex [20]. The natural concentration of DHEA in the blood is around 10 µM in young adults. It has been reported that the DHEA concentration in the body decreases with age [21]. This decline in DHEA suggests that a relative deficiency of this steroid may be causally related to the development of age-related illnesses such as atherosclerosis, metabolic diseases,　bone loss, cancer, and cognitive decline [22, 23]. It has been reported that DHEA has antioxidant properties and its protective effects have the potential to improve various diseases such as DM, osteoporosis, arterial sclerosis, Alzheimer’s disease, immune function, and adrenal insufficiency [21–25]. Recent double-blind, placebo-controlled studies have shown that DHEA replacement therapy in the elderly improves muscle strength, bone density, and depression [26–28]. In addition, DHEA treatment suppresses oxidative stress in the rabbit renal cortex by inhibiting NOX activity [29]. However, the antioxidant effects of DHEA on tenocytes and tendons have not been reported and remain unknown. We hypothesized that DHEA might be effective against diabetic tendinopathy by suppressing NOX. Thus, the purpose of this study was to evaluate the in vitro and in vivo antioxidant effects of DHEA on NOX-derived oxidative stress under hyperglycemic conditions on tenocytes and tendons.

## Methods

All animal procedures were performed with the approval and guidance of the Animal Care and Use Committee of our institution. The experiments were conducted in accordance with the ARRIVE guidelines.

### In vitro experiments

#### Cell preparation

The Achilles tendons were excised from 15 healthy male Sprague-Dawley (SD) rats of eight weeks of age. Tendons were washed twice with phosphate-buffered saline (PBS) and cut into small pieces measuring approximately 1.5 to 2.0 mm^3^. Several pieces were placed on a culture plate and cultured in Dulbecco’s modified Eagle’s medium (DMEM) (Sigma-Aldrich, St. Louis, MO, USA) supplemented with 10 % fetal bovine serum (FBS), 100 µg/mL streptomycin, and 100 U/mL penicillin. The explants were incubated at 37 °C in a humidified atmosphere of 5 % CO_2_/95 % air. Once the tenocytes migrating from the explants had attained a subconfluent state, the cells were subcultured after trypsin digestion [18, 19, 30]. The culture medium was changed every five days. Cells from passages 2 to 3 were used in this study.

#### Cell proliferation assays

Cell proliferation was measured by a water-soluble tetrazolium salt (WST) assay using a Cell Counting Kit-8 (Dojindo, Kumamoto, Japan). A total of 5000 cells were seeded in 100 µL DMEM in each well of a 96-well plate. Cells were cultured for 24 h in a CO_2_ incubator at 37 °C before the WST assay evaluation and then incubated for another 48 h in DMEM containing four different DHEA (Tokyo Chemical Industry, Tokyo, Japan) concentrations (0 as a control, 1, 10, 20, and 50　µM). DHEA was dissolved in 0.1 % dimethyl sulfoxide (DMSO). For the WST assay, each well was supplemented with 10 µL WST for four hours at 37 °C in a CO_2_ incubator before spectrophotometric evaluation at 450 nm (*n* = 15 per group).

#### Experimental protocol

Tenocytes were seeded onto 12-well culture plates at a seeding density of 10^5^ cells per well and incubated in DMEM with two different glucose concentrations: 6 mM in the low-glucose (LG) group and 33 mM in the high-glucose (HG) group, according to a previous study [31]. DHEA was first dissolved in DMSO to obtain a 2 mM stock solution and then diluted to a final concentration of 10 µM based on a previous study using muscle cells [21].　DHEA was added at cell seeding, and the tenocytes　were divided into four groups: the control group (LG DHEA-), LG with DHEA (LG DHEA+), HG without DHEA (HG DHEA-), and HG with DHEA (HG DHEA+) (*n* = 12 per group). The same amount of DMSO was added to all groups (*n* = 15 per group).

#### Quantitative real-time polymerase chain reaction (PCR) analysis

At 48 h, total RNA from tenocytes was extracted using an RNeasy Mini Kit (Qiagen, Valencia, CA, USA). Using a High Capacity cDNA Reverse Transcription Kit (Applied Biosystems, Foster City, CA, USA), total RNA was reverse-transcribed into single-strand complementary DNA (cDNA). PCR in triplicate was performed on the cDNA with a 7900HT Fast Real-Time PCR System and SYBR Green reagents (Applied Biosystems). The messenger RNA (mRNA) levels of NOX1, NOX4, and IL-6 were analyzed. The primer sequences are shown in Table 1. Results were normalized to the mRNA levels of the housekeeping gene glyceraldehyde 3-phosphate dehydrogenase (GAPDH)　and expressed relative to their levels in the control culture using the 2^−ΔΔCt^ method [32] as previous studies [18, 19] (n = 15 per group).

**Table 1:** Primer sequences used for polymerase chain reaction

Gene	Oligonucleotide sequence
NOX1	Forward 5’ GTGGCTTTGGTTCTCATGGT 3’ Reverse 5’ TGAGGACTCCTGCAACTCCT 3’
NOX4	Forward 5’ GGGCCTAGGATTGTGTTTGA 3’ Reverse 5’ CTGAGAAGTTCAGGGCGTTC 3’
Type I collagen	Forward 5’ TGGAGACAGGTCAGACCTG 3’ Reverse 5’ TATTCGATGACTGTCTTGCC 3’
Type III collagen	Forward 5’ TAAAGGGTGAACGGGGCAGT 3’ Reverse 5’ ACGTTCCCCATTATGGCCAC 3’
MMP-2	Forward 5’ GGAAGCATCAAATCGGACTG 3’ Reverse 5’ GGGCGGGAGAAAGTAGCA 3’
TIMP-1	Forward 5’ ATAGTGCTGGCTGTGGGGTGTG 3’ Reverse 5’ TGATCGCTCTGGTAGCCCTTCTC 3’
TIMP-2	Forward 5’ GGACACGCTTAGCATCACCCAGA 3’ Reverse 5’ GTCCATCCAGAGGCACTCATCC 3’
IL-6	Forward 5’ GGTCTTCTGGAGTTCCGTTTC 3’ Reverse 5’ GGTCTTGGTCCTTAGCCATCT 3’
GAPDH	Forward 5’ GGTGGTCTCCTCTGACTTCAACA 3’ Reverse 5’ GTTGCTGTAGCCAAATTCGTTGT 3’

#### Detection of ROS accumulation

According to previous reports [18, 19], intracellular ROS levels in tenocytes of each group were detected by the oxidation-sensitive fluorescent probe dichloro-dihydro-fluorescein diacetate (DCFH-DA) using the Total ROS/Superoxide Detection Kit (Enzo Life Science, Farmingdale, NY, USA) following the manufacturer’s protocol. Tenocytes (5 × 10^4^) were incubated with DCFH-DA at a final concentration of 10 µM for 60 min at 37 °C in the dark, washed three times with PBS, trypsinized, and resuspended. For quantification, the number of ROS-positive cells and DAPI-positive cells in four rectangular areas (0.75 mm × 1.0 mm) in each slide were counted, and the mean values were calculated. Each area was randomly selected and the cells were manually counted by two blinded investigators. The percentage of ROS-positive cells was calculated using the formula (number of ROS-positive nuclei/number of DAPI-positive nuclei) × 100 and expressed as the mean of the four areas (*n* = 15 per group).

#### Immunofluorescence staining for analysis of apoptotic cells

According to previous studies [18, 19], nuclear fragmentation was detected by terminal deoxynucleotidyl transferase dUTP nick end labeling (TUNEL) staining with an APO-DIRECT Kit (Phoenix Flow Systems, San Diego, CA, USA) according to the manufacturer’s protocol, using fixed cells (4 % paraformaldehyde/PBS) with 2-(4-amidinophenyl)-1 H-indole-6-carboxamidine (DAPI). For quantification, the number of apoptosis-positive and DAPI-positive cells in four rectangular areas (0.75 mm × 1.0 mm) in each slide was counted, and the mean values were calculated. Each area was randomly selected and the cells were manually counted by two blinded investigators. The percentage of apoptosis-positive cells was calculated using the formula (number of apoptosis-positive nuclei/number of DAPI-positive nuclei) × 100 and expressed as a mean of the four areas (*n* = 15 per group).

### In vivo animal experiments

#### Type I diabetic rat model

To induce DM, a single dose of streptozotocin (STZ; 65 mg/kg; Sigma-Aldrich) dissolved in sodium citrate buffer (pH 4.5) was intravenously administered to 18 eight-week-old healthy male SD [33]. Following the injections, all animals were housed in standard cages with unrestricted access to food, water, and activity. All STZ-injected rats became diabetic ten days after STZ injection. Their mean blood glucose level was 405.8 ± 62.5 mg/mL (mean ± standard deviation), while that of healthy control rats was < 150 mg/dL [33].

#### Experimental protocol

DM rats were randomly divided into two groups: a control group and a DHEA-injected group (DHEA group) (*n* = 9 in each group). In the DHEA group, two weeks after STZ injection, 50 mg/kg DHEA [34, 35] and vehicle (10 % DMSO) were injected intraperitoneally every other day for 4 weeks [36–38]. In the control group, vehicle alone was injected in the same manner. The animals were sacrificed 4 weeks after the first DHEA or vehicle injection (6 weeks after STZ injection), according to a previous study [18]. The right Achilles tendon was used for immunohistological evaluation, and the left was used for quantitative real-time PCR.

#### Achilles tendon histology and NOX immunohistochemistry analysis

For immunohistological analysis, hematoxylin and eosin (H&E) staining and staining for NOX1 and NOX4 were performed using the nine right tendons of diabetic rats from each group according to a previous study [18]. Frozen, long-axis sections of Achilles tendons were sectioned into 7-µm-thick specimens and fixed using 10 % phosphate-buffered paraformaldehyde at room temperature. Histological evaluation of fiber structure and arrangement, nuclear morphology, and zonal variations in tendon cellularity was performed using H&E staining [39]. Each variable was scored between 0 and 3; 0 being normal, 1 slightly abnormal, 2 abnormal, and 3 markedly abnormal [39]. The grading of H&E-stained sections was performed in five randomly selected optical fields in each section and evaluated by two blinded investigators.

The immunohistochemical evaluation of NOX expression was performed by using anti-NOX1 and anti-NOX4 antibodies (Abcam, Cambridge, UK). Sections were incubated with proteinase for 10 minutes, treated with 3 % hydrogen peroxide (Wako Pure Chemical Industries, Osaka, Japan) to block endogenous peroxidase activity, and incubated with anti-NOX1 or anti-NOX4 antibodies (1:100 for both) at 4°C overnight [18]. Then, sections were incubated with a peroxidase-labeled immunoglobulin antibody (Nichirei Bioscience, Tokyo, Japan) at room temperature for 30 minutes [18]. The signal (NOX1 and NOX4) was detected by the formation of a brown color following incubation with the peroxidase substrate 3,3’-diaminobenzidine (Nichirei Bioscience). Sections were counterstained with hematoxylin and examined microscopically. For semi-quantitative analysis, the ratio of NOX-positive tendon cells per field was determined in five randomly selected fields for each tissue section by two blinded investigators [18].

#### Quantitative real-time PCR analysis

Nine left tendons from each group were used for quantitative real-time PCR. The Achilles tendons were cut into small pieces and minced. Isolated Achilles tendons were enzymatically dissociated with type II collagenase (Worthington Biochemical Corporation, Lakewood, NJ, USA) and prepared for RNA isolation [32]. Total RNA was extracted using an RNeasy Mini Kit. Reverse transcription into single-stranded cDNA and real-time PCR was performed as previously described. Expression of NOX1, NOX4, IL-6, MMP-2, TIMP-2, and type I and III collagen (col1 and col3) was evaluated as previously described.

### Statistical analysis

All data are expressed as means and standard deviations. All statistical analyses of recorded data were　performed using the Excel statistical software package (Ekuseru-Toukei 2015; Social Survey Research Information Co., Ltd., Tokyo, Japan). Comparisons between more than two groups were performed by two-way ANOVA and Tukey’s post-hoc test. Comparisons between two groups were performed using Student’s *t* test. A *p*-value < 0.05 was considered statistically significant.

## Results

### In vitro experiments

#### Cell proliferation assays

**Fig. 1:**
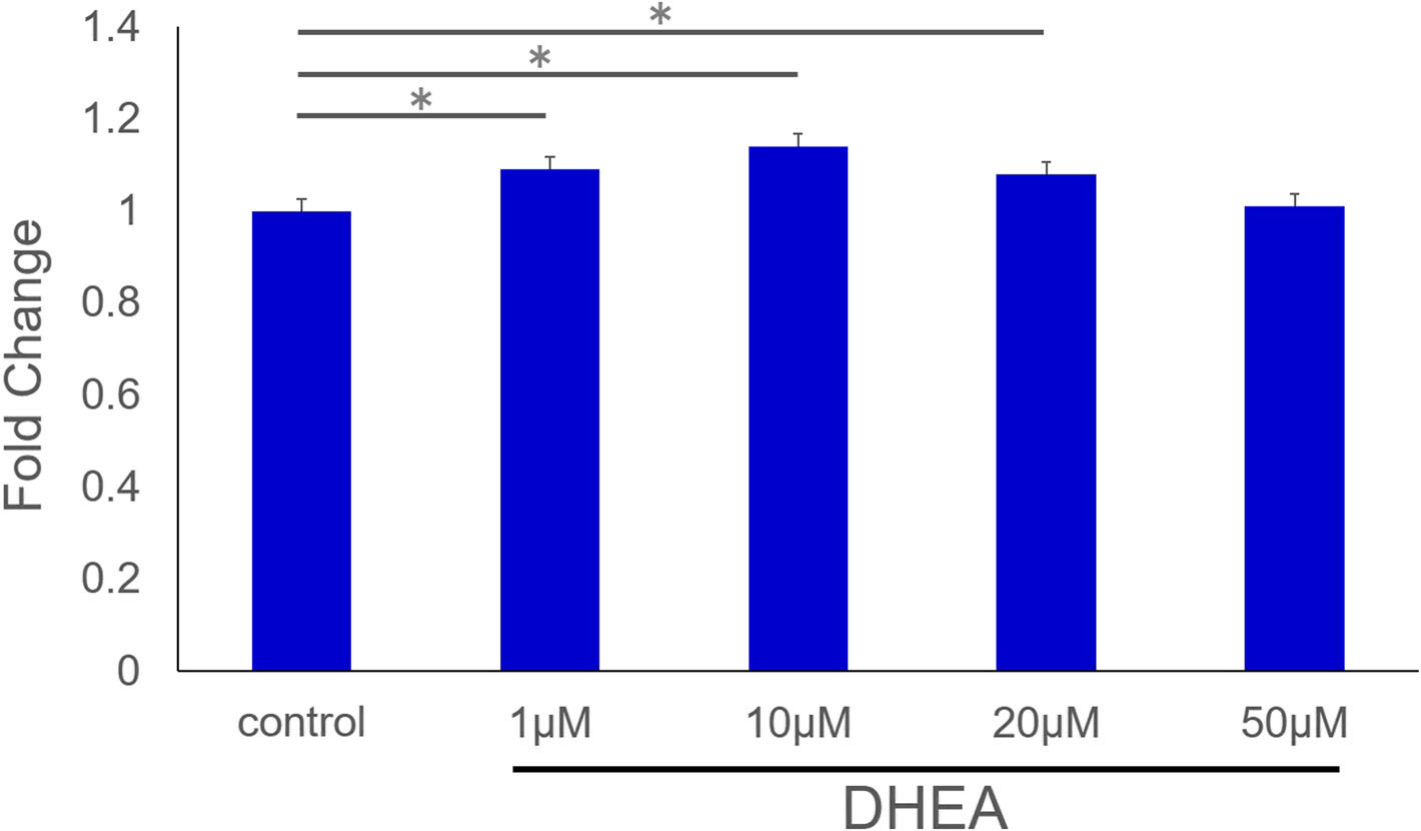
Cell proliferation. Cell proliferation in the 1, 10, and 20 µM Dehydroepiandrosterone (DHEA) groups was significantly higher than that in the control group. (*p < 0.05). Proliferation in the 20 and 50 µM DHEA groups was lower in a dose-dependent manner

The WST assay of tendon cell proliferation showed that the 1, 10, and 20 µM DHEA groups had significantly enhanced cell viability compared to the control group (*p* < 0.05), and the viability of the 10 µM DHEA group was the highest among all groups. The 20 and 50 µM DHEA groups were lower in a dose-dependent manner (Fig. 1).

#### Quantitative real-time PCR analysis

**Fig. 2:**
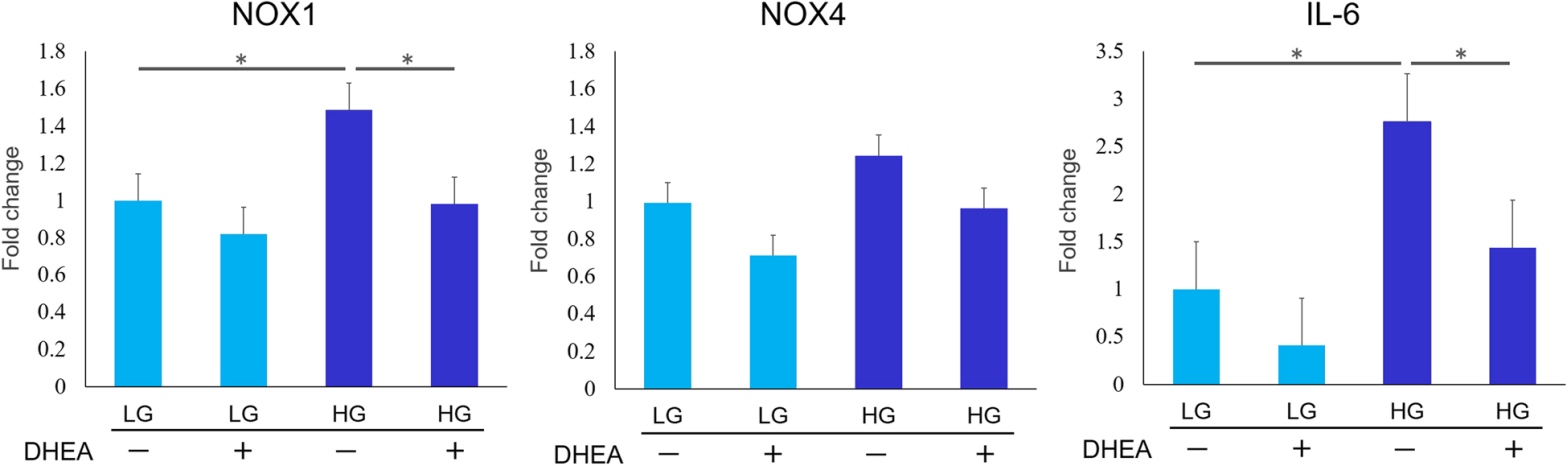
Quantitative real-time PCR analysis (in vitro) . Messenger RNA (mRNA) expression of NADPH oxidase (NOX1) and interleukin-6 (IL-6) in the high-glucose without dehydroepiandrosterone (HG DHEA-) group were significantly higher than those in the low-glucose without DHEA (LG DHEA-) group at 48 h. (*p < 0.05). The expression of NOX1 and IL-6 in the high-glucose with DHEA group (HG DHEA+) were significantly lower than those in HG DHEA– group. (*p < 0.05). There was no significant difference in NOX4 expression between the groups

At 48 h, NOX1 and IL-6 mRNA expression in the HG DHEA- group was significantly higher than in the LG DHEA- group (*p* < 0.05). NOX1 and IL-6 mRNA expression in the HG DHEA + group was significantly lower than in the HG DHEA- group (*p* < 0.05). There was no significant difference in the expression of NOX4 mRNA within each group (Fig. 2).

#### ROS accumulation

**Fig. 3:**
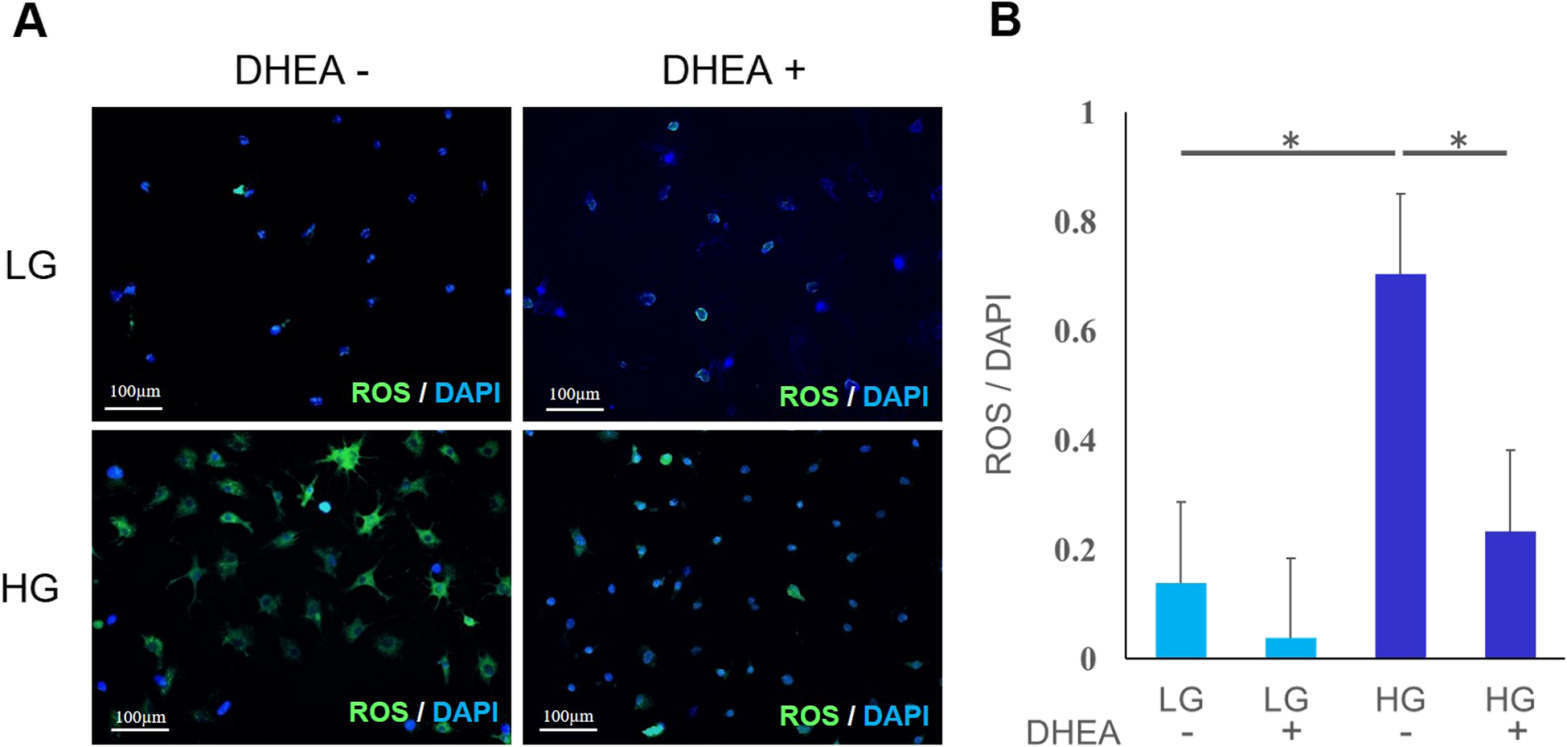
ROS accumulation . (**A**) Fluorescence staining showing reactive oxygen species (ROS) accumulation (green) in tenocytes and nuclei (4’ 6-diamidino-2-phenylindole, DAPI) (blue). There was lower ROS accumulation in the low-glucose without dehydroepiandrosterone (LG DHEA–) and with DHEA (LG DHEA+) groups. Increased ROS accumulation observed in the high-glucose (HG) groups, with the HG DHEA + being lower than the HG DHEA– group. (**B**) Reactive oxygen species (ROS) accumulation was analyzed by fluorescence intensity normalized to cell number. The ROS accumulation in the high-glucose without dehydroepiandrosterone (HG DHEA–) group was greater than that in the low-glucose without DHEA (LG DHEA–) at 48 h. The ROS accumulation in the HG DHEA + group was significantly smaller than that in the HG DHEA– group. *p < 0.05. DAPI, 4’,6-diamidino-2-phenylindole

The cytoplasm of ROS-positive cells was stained green (Fig. 3 A). A quantitative analysis of ROS-positive cells is shown in Fig. 3B. The ROS accumulation in the HG DHEA- group was significantly greater than that of the LG DHEA- group (*p* < 0.05). Accumulation in the HG DHEA + group was significantly lower than that of the HG DHEA- group (*p* < 0.05). There was no difference between the LG groups (Fig. 3B).

#### Immunofluorescence staining for analysis of apoptotic cells

**Fig. 4:**
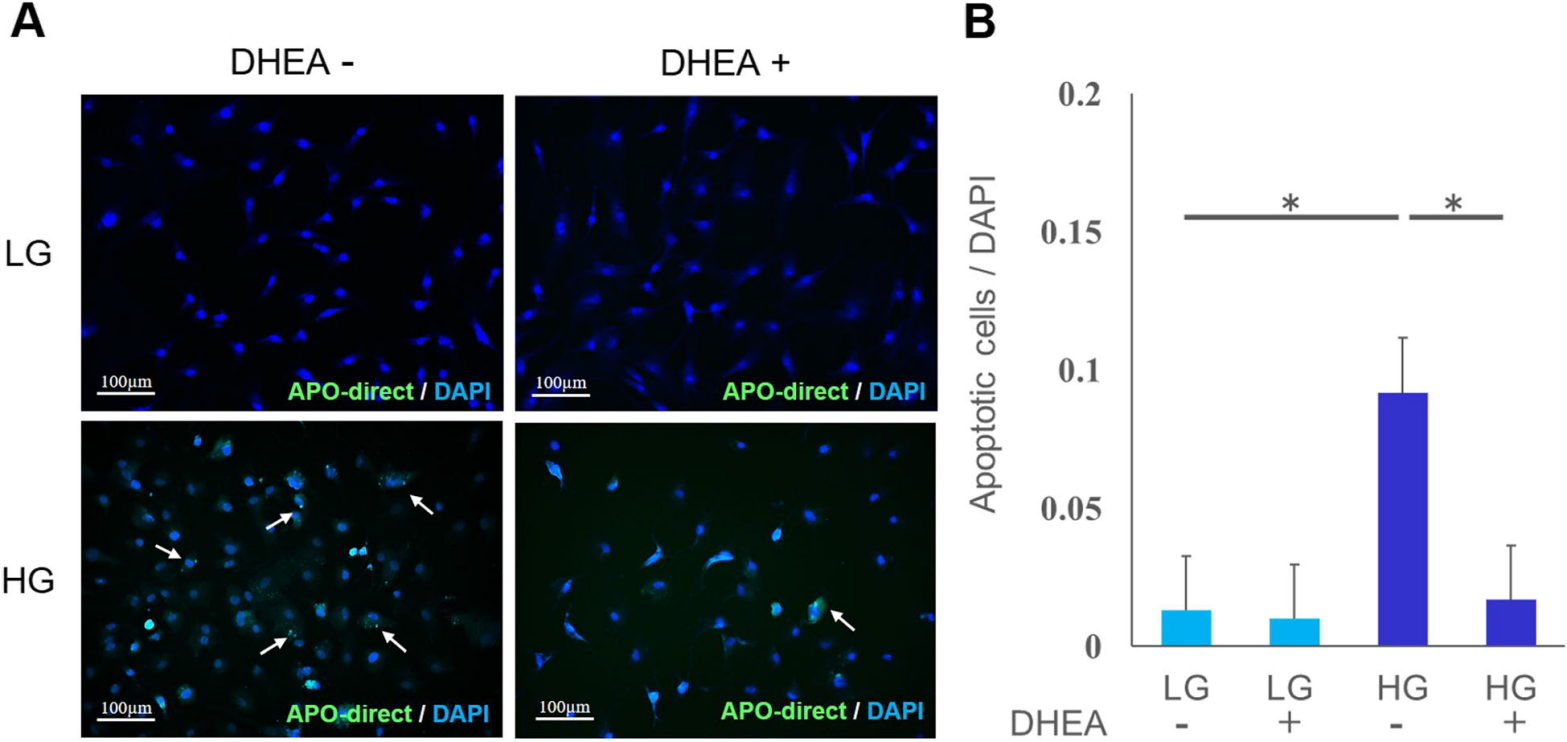
Immunofluorescence staining for analysis of apoptotic cells. (**A**) Immunofluorescence staining showing all cells (blue; DAPI) and apoptotic cells (green; APO-direct, white arrow (→)). There were few apoptotic cells in the low-glucose without dehydroepiandrosterone (LG DHEA–) and with DHEA (LG DHEA+) groups. There was induction of apoptosis in the high-glucose (HG) groups. The number of apoptotic cells in the HG DHEA + group was lower than that in the HG DHEA– group. (**B**) The number of apoptotic cells was analyzed by fluorescence intensity normalized to cell number. The number of apoptotic cells in the high-glucose without dehydroepiandrosterone (HG DHEA–) group was significantly higher than that in low-glucose without DHEA (LG DHEA–) at 48 hours. The number of apoptotic cells in HG DHEA + group was significantly decreased compared with that in HG DHEA– group. *p < 0.05. DAPI, 4’,6-diamidino-2-phenylindole

Apoptotic cells were observed in both HG groups, and abnormal nuclear morphology such as nuclear fragmentation was found in apoptotic cells (Fig. 4 A). A quantitative analysis of apoptotic cells is shown in Fig. 4B. The number of apoptotic cells in the HG DHEA + group was lower than that in the HG DHEA– group, and there was a statistically significant difference between the two groups (*p* < 0.05) (Fig. 4B).

### In vivo animal experiments

#### Achilles tendon histology and immunohistochemistry for NOX analysis

**Fig. 5:**
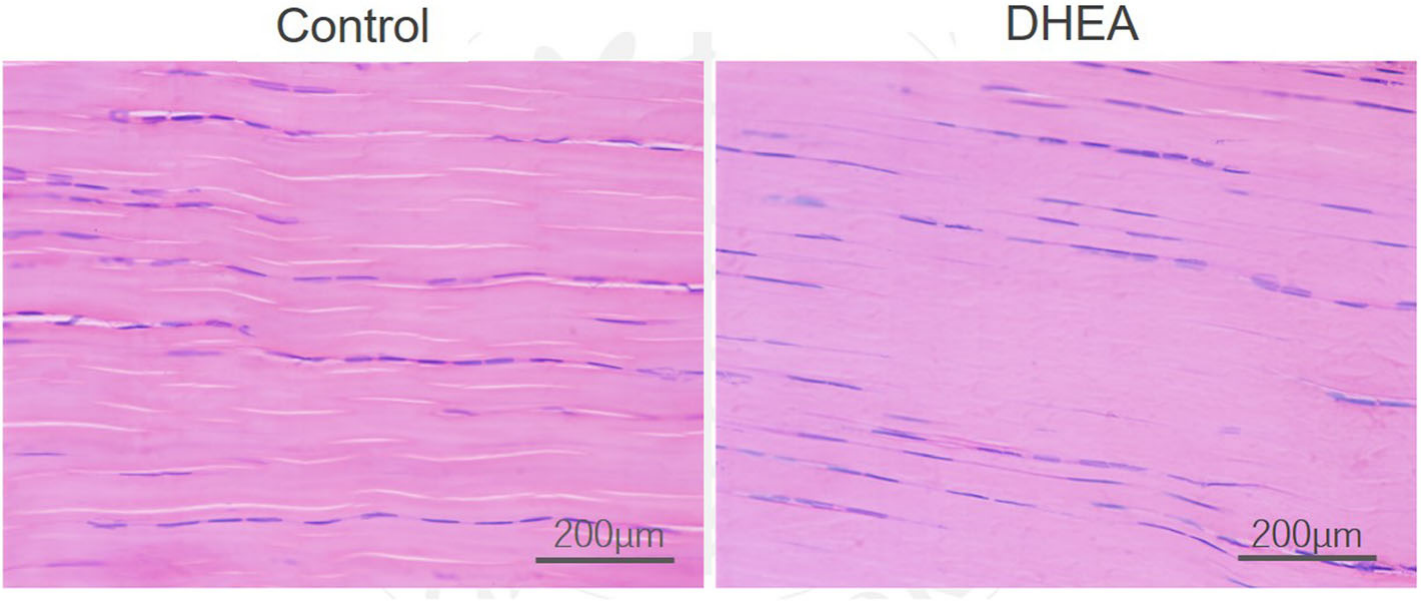
Hematoxylin and eosin staining of diabetic rat Achilles tendon. Hematoxylin and eosin (H&E) staining of diabetic rat Achilles tendons in the control and dehydroepiandrosterone (DHEA) groups harvested at six weeks following streptozotocin treatment. No obvious pathological difference between groups was observed

Histological evaluation showed no significant difference in fiber structure and arrangement, rounding of the nuclei, and regional variations in cellularity between the control and DHEA groups (Table 2). The fiber structure and arrangement showed near parallel collagen fiber orientation and flattened or spindle-shaped nuclei arranged in rows between the collagen fibers in both groups (Fig. 5).

**Fig. 6:**
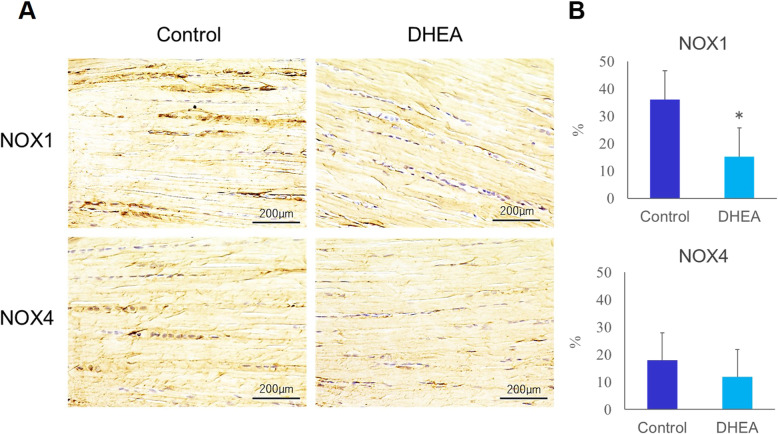
Immunohistochemical staining for NOX1 and NOX4 expression. (**A**) Immunohistochemical staining for NADPH oxidase (NOX)1 and NOX4 expression (brown) in the diabetic rat Achilles tendon. Increased expression of NOX1 in Achilles tendon was observed in dehydroepiandrosterone (DHEA) group compared with control group. There was no difference in the expression of NOX4 between DHEA and control group. (**B**) Semi-quantitative analysis of cells positive for NADPH oxidase (NOX)1 (left) and NOX4 (right). The ratio of NOX1-positive cells in diabetic rat Achilles tendon was significantly higher in dehydroepiandrosterone (DHEA) group than in control group. (*p < 0.05). No significant difference was observed between control and DHEA group in the ratio of NOX4-positive cells

Immunohistochemical staining of the Achilles tendon at four weeks following DHEA injection showed that NOX1 expression markedly decreased within the tenocytes of diabetic rats with DHEA (Fig. 6 A). NOX4 was weakly expressed in both groups and showed no difference between the two groups (Fig. 6 A). Using semi-quantitative analysis, the percentage of NOX1-positive cells was significantly lower in the Achilles tendon of the DHEA group (*p* < 0.05) (Fig. 6B). There was no significant difference in the percentage of NOX4-positive cells between the groups (*p* < 0.05) (Fig. 6B).

**Table 2:** Tendon pathological scores

	Mean control (SD)	Mean DHEA (SD)	*p*-value *
**Fiber structure**	0.89 (0.60)	0.74 (0.57)	0.21
**Fiber arrangement**	0.91 (0.54)	0.83 (0.60)	0.47
**Nuclear morphological changes (rounding)**	0.67 (0.59)	0.52 (0.50)	0.19
**Regional variations in cellularity**	0.45 (0.49)	0.39 (0.49)	0.53

* Student’s *t*-test

*n* = 9 rats in the control group, *n* = 9 rats in the DHEA group

#### Quantitative real-time PCR

**Fig. 7:**
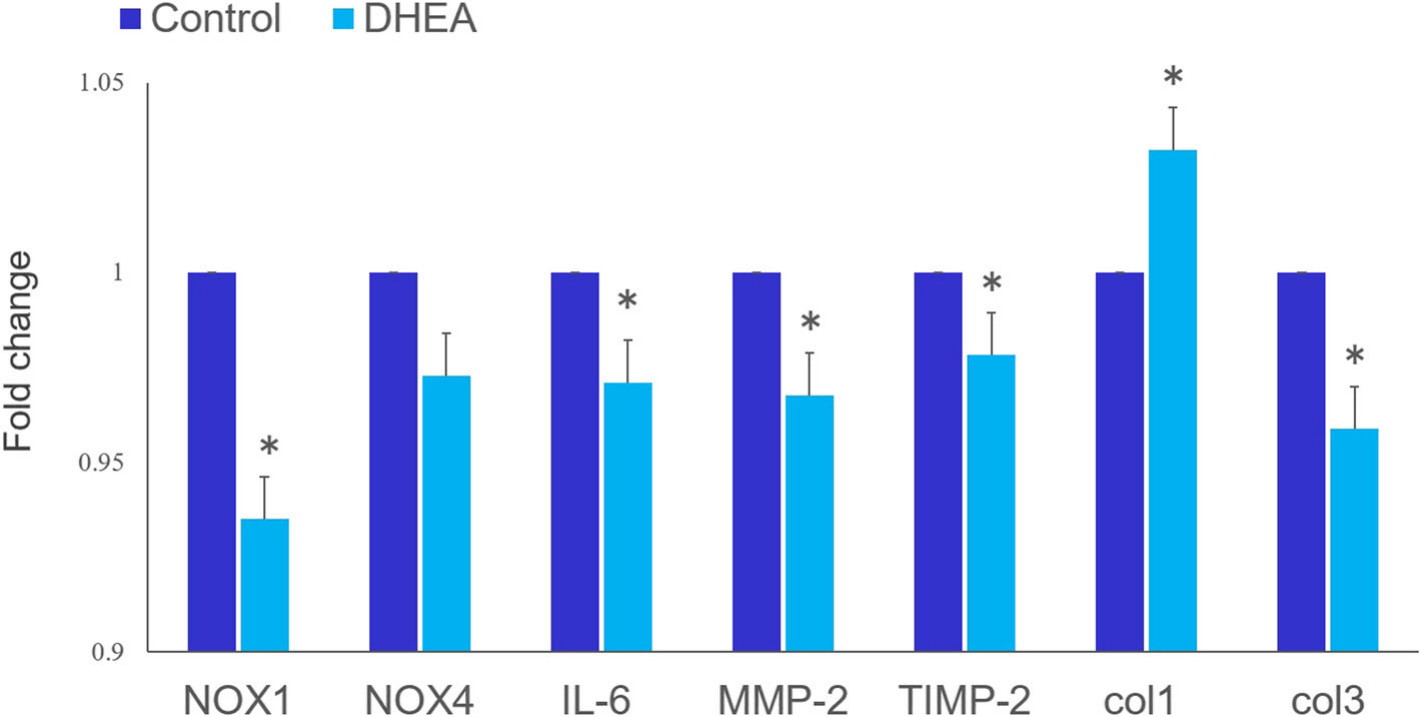
Quantitative real-time PCR analysis (in vivo). Relative fold changes in messenger RNA (mRNA) levels in the tendon. The mRNA expressions of NADPH oxidase (NOX)1, interleukin (IL)-6, matrix metalloproteinase (MMP)-2, tissue inhibitors of matrix metalloproteinase (TIMP)-2, and type III collagen (col3) in diabetic rat Achilles tendons were significantly lower in the dehydroepiandrosterone (DHEA) group than in the control group. (*p < 0.05). No significant difference between control and diabetic rats was seen in the expression of NOX4. The expression of type I collagen (col1) was significantly higher in the DHEA group than in the control group

The expression of NOX1 in the Achilles tendons was significantly lower in the DHEA group (*p* < 0.05). There was no significant difference in NOX4 expression between the control and DHEA groups (Fig. 7). The expression of IL-6, MMP-2, TIMP-2, and col3 was significantly lower in the DHEA group (*p* < 0.05), while the expression of col1 was significantly higher in the DHEA group (*p* < 0.05) (Fig. 7).

## Discussion

DHEA is a circulating steroid hormone abundantly produced by the mammalian adrenal cortex [20]. DHEA has the potential to improve the disease status of conditions such as DM, osteoporosis, arterial sclerosis, Alzheimer’s disease, and adrenal insufficiency due to its antioxidant effects [16, 17]. In the field of orthopedics, DHEA has been shown to exert beneficial effects on osteoarthritis-damaged cartilage by actively regulating the balance of anabolic and catabolic factors (e.g., MMPs/TIMP-1 and ADAMTS/TIMP-3) and inhibiting catabolic signaling pathways (e.g., Wnt/β-catenin) [40]. Although there are many studies regarding the effects of DHEA on knee osteoarthritis, there are no reports concerning the effectiveness of DHEA in preventing and treating tendon disease.

Previous studies in rat tenocytes and Achilles tendon have shown that high glucose conditions upregulate the expression of NOX1 mRNA and ROS production [18, 19]. Therefore, overexpression of NOX could be a potential therapeutic target for diabetic tendinopathy. Here, the in vitro effectiveness of DHEA on hyperglycemia-induced oxidative stress in rat tenocytes was investigated. Our results showed that DHEA enhanced the viability of tenocytes, had no cytotoxic effects as reported in muscle cells [21], and suppressed the expression of NOX1, the overproduction of ROS, and apoptosis. Additionally, our in vivo studies also showed that DHEA reduced NOX1 expression (mRNA and protein) in the diabetic rat Achilles tendon.

The antioxidant effects of DHEA are multi-targeted, and several explanations have been proposed, including inhibition of glucose-6-phosphate dehydrogenase (G6PD) [41], alteration of catalase expression [42], upregulation of the redox system [43], change in fatty acid composition of cell membranes, and cytokine production [24]. The existence of a high-affinity DHEA receptor-activated phosphatidyl-inositol 3-kinase/Akt pathway has also been shown [44]. However, the detailed mechanism remains unclear. Moreover, there is debate as to whether the effects of DHEA are owing to DHEA itself, its metabolites, or a combination of the two. Huerta-García et al. demonstrated the protective effects of DHEA against oxidative stress induced by high concentrations of glucose in endothelial cells [45]. Kiersztan et al. reported that DHEA reduced oxidative stress in the kidney-cortex due to a decline in NOX activity [29]. In the present study, the mechanism of DHEA on tenocytes was not fully elucidated; however, DHEA showed antioxidant effects by inhibiting NOX1 expression against high glucose-induced oxidative stress, and the results were consistent with previous reports. This suggests that DHEA may be effective as a preventive medicine for diabetic tendon disorders.

It was reported that no significant change was observed in the histological evaluation of diabetic rat Achilles tendon at 6 weeks after STZ administration compared with normal SD rats [18, 46], and similar results were obtained in this study. These results were thought to be due to the evaluation of the initial response of diabetic tendinopathy [18, 46].

A systematic review of cytokines in tendon disease reported that the expression of IL-1β, IL-6, and TNF-α in animal tendon injury models tended to increase from the early phase of tendon healing [18]. IL-6 was the only cytokine involved in human tendon disease and elevated in tendon tears [47]. A previous study showed a higher expression of IL-6 in rat tenocytes and Achilles tendons under high-glucose conditions and indicated that high-glucose conditions might stimulate inflammatory processes within the tendon [18]. Additionally, type III collagen is expressed during inflammatory processes and the early phase of the healing process, although approximately 90 % of the collagen in normal tendons is type I [48]. Our in vitro and in vivo studies showed that DHEA exerted anti-inflammatory effects by decreasing IL-6 expression in rat tenocytes and Achilles tendons. In addition, the decrease in type III collagen and increase in type I collagen further confirmed the anti-inflammatory effects of DHEA and suggested that the healing process requiring the expression of type III collagen may not have occurred because of the protective effects of DHEA.

The balance between MMP and TIMP expression regulates normal tendon metabolic activity [49]. During inflammation, MMPs cleave damaged interstitial collagen for remodeling, while TIMPs inhibit the overexpression of MMPs [49]. In a rabbit supraspinatus tendon tear model, MMP-2 and TIMP2 were expressed and activated during the healing process, suggesting that they play an important role in the remodeling process [50].　 In the diabetic rat Achilles tendon, it has been reported that MMP-2 and TIMP-2 were also increased as in acute tendon tear, and tendon matrix synthesis and turnover were enhanced under hyperglycemic conditions [18]. In the present study, DHEA significantly decreased the expression of MMP-2 and TIMP-2 in the Achilles tendon of diabetic rats. This suggests that DHEA may prevent the development of conditions requiring a healing process, suppress the overexpression of MMP-2 through its anti-inflammatory response, and improve tendon matrix synthesis and turnover, which were enhanced by hyperglycemia.

This study had several limitations. First, the monolayer culture of tenocytes in vitro does not reproduce true physiological conditions. However, previous studies have demonstrated that primary tenocytes maintained phenotypical stability until passage 5 when passaged in subconfluence [51, 52]. Isolation of cells from the native tendon was performed according to previously reported and accepted methods [30]. Second, our in vivo experiments only evaluated the early changes in diabetic tendinopathy; therefore, changes in fibril organization were not observed. Further research is needed on the long-term effects of DHEA. Third, the biomechanical properties of DHEA on the Achilles tendon in diabetic rats were not investigated due to the small number of experimental animals used. Fourth, our results indicated that DHEA has antioxidant and anti-inflammatory properties in tenocytes and tendons under hyperglycemic conditions. However, the clinical efficacy of DHEA supplementation　in humans to reduce pathophysiological symptoms related to diabetic tendinopathy remains to be evaluated.

## Conclusions

DHEA showed antioxidant and anti-inflammatory effects by reducing the expression of NOX1 and IL-6, the overproduction of ROS, and the suppression of apoptosis in our experimental model. Additionally, DHEA improved tendon matrix synthesis and turnover by reducing the expression of MMP-2/TIMP-2 and type III collagen induced by hyperglycemic conditions. These results suggest that DHEA could be a preventive drug for diabetic tendinopathy.

## Data Availability

The datasets generated during and analyzed during the current study are not publicly available due to the inclusion of unpublished data but are available from the corresponding author on reasonable request.

## Abbreviations

DM: Diabetes mellitus
ROS: Reactive oxygen species
NOX: NADPH oxidase
MMP: Matrix metalloproteinase
TIMP: Tissue inhibitors of matrix metalloproteinase
DHEA: Dehydroepiandrosteron.
SD: Sprague-Dawley
PBS: Phosphate-buffered saline
DMEM: Dulbecco’s Modified Eagle’s Medium
FBS: Fetal bovine serum
PCR: Polymerase chain reaction
STZ: Streptozotocin
DMSO: Dimethyl sulfoxide
H&E: Hematoxylin and eosin.
